# MicroRNAs-Dependent Regulation of PPARs in Metabolic Diseases and Cancers

**DOI:** 10.1155/2017/7058424

**Published:** 2017-01-12

**Authors:** Dorothea Portius, Cyril Sobolewski, Michelangelo Foti

**Affiliations:** Department of Cell Physiology and Metabolism and Diabetes Center, Faculty of Medicine, University of Geneva, Geneva, Switzerland

## Abstract

Peroxisome proliferator-activated receptors (PPARs) are a family of ligand-dependent nuclear receptors, which control the transcription of genes involved in energy homeostasis and inflammation and cell proliferation/differentiation. Alterations of PPARs' expression and/or activity are commonly associated with metabolic disorders occurring with obesity, type 2 diabetes, and fatty liver disease, as well as with inflammation and cancer. Emerging evidence now indicates that microRNAs (miRNAs), a family of small noncoding RNAs, which fine-tune gene expression, play a significant role in the pathophysiological mechanisms regulating the expression and activity of PPARs. Herein, the regulation of PPARs by miRNAs is reviewed in the context of metabolic disorders, inflammation, and cancer. The reciprocal control of miRNAs expression by PPARs, as well as the therapeutic potential of modulating PPAR expression/activity by pharmacological compounds targeting miRNA, is also discussed.

## 1. Introduction

Peroxisome proliferator-activated receptors (PPARs) are a family of nuclear receptors involved in various biological functions but with a prominent role in metabolic homeostasis of carbohydrates and lipids [1]. The three PPAR isoforms, PPAR*α* (NR1C1), PPAR*β*/*δ* (NR1C2), and PPAR*γ* (NR1C3), share 60% to 80% of structural homology [2, 3] and exhibit a distinct tissue expression pattern but can exert similar or different physiological functions [3]. In the canonical model, PPARs are activated in the cytoplasm by specific ligands [1–6] and then translocate into the nucleus, where they form a complex predominantly with the nuclear receptor Retinoid-X-Receptor (RXR), to transactivate gene expression by binding to PPAR response elements (PPREs) on gene promoters [6, 7]. In contrast, noncanonical PPAR activity suppresses gene transcription through direct protein-protein interactions with other transcription factors, for example, the nuclear factor-kB (NFkB) or activated protein-1 (AP-1) [1, 3]. PPARs activity is also tightly dependent on the binding of other cofactors such as PGC1*α* (peroxisome proliferator-activated receptor coactivator-1*α*) and p300 or CREB binding protein—or on the contrary on the binding of corepressor proteins, for example, NCOR (nuclear receptor corepressor) or SMRT (silencing mediator for retinoid and thyroid hormone receptor), which hamper PPARs interactions with PPRE [3].

Through complex regulatory mechanisms, PPARs exert a tight control on energy homeostasis by modulating the expression of key genes involved in lipid metabolism [5, 6], adipocytes differentiation [5], and carbohydrate metabolism [5, 6]. The implication of PPARs in inflammatory processes and specific cancers is further suggested by recent studies (reviewed in [3, 8, 9]). These key and pleiotropic roles of PPARs in cellular processes have led to the development of pharmacologic agonists, for example, thiazolidinediones and fibrates [10, 11], to treat metabolic disorders or other diseases such as atherosclerosis [2, 5, 12]. However, long-term treatment with PPARs agonists triggers uncontrolled side effects in patients (e.g., oedema, weight gain, heart failure, and bone fractures) and in some cases they may even promote tumorigenesis [6, 8, 13]. Alternative therapeutic options to control distinct PPARs activities in specific tissues are therefore desirable but require that we deepen our understanding of the molecular mechanisms controlling PPARs expression/activity in diseases.

Recently, a wealth of studies has suggested that epigenetic mechanisms, for example, DNA methylation, histone modifications, or small noncoding RNA (i.e., microRNAs), importantly affect physiological or pathological mechanisms involved in a wide variety of diseases and cancers. In the case of PPARs, methylation of their promoters [14, 15], or histone acetylation [16], has been reported to affect PPARs expression and physiological processes under their control. More recently, other epigenetic alterations, in particular those leading to abnormal microRNAs (miRNAs) expression, have also been implicated in the regulation of PPARs expression or activity [17]. Indeed several miRNAs were reported to either directly target PPARs mRNA or to indirectly affect their expression/activities by targeting PPARs-associated cofactors and repressors, thus providing a further level of complexity in these regulatory mechanisms [18–20].

In this review, we discuss the current knowledge about miRNAs-dependent regulation of PPARs and their cofactors in physiological and pathological processes. Most of available studies dealing with this topic are restrained to metabolic diseases (e.g., diabetes, fatty liver diseases, and cardiovascular diseases) and associated cancers (e.g., liver cancers) in tissues where the role of PPARs is well characterized (e.g., liver, adipose tissue, muscles, and heart). Other rare studies investigating PPARs regulation by miRNAs in different tissues (e.g., bone marrow, neurons, and cartilage) or type of cancers (e.g., neuroblastoma, prostate cancer), unrelated to metabolic disorders, are also considered. Finally, the reciprocal regulation of specific miRNAs by PPARs, as well as potential miRNA-based pharmacological approaches to therapeutically modulate PPARs expression and/or activity, was also examined.

## 2. miRNAs

MicroRNAs (miRNAs) are endogenous small noncoding RNAs of approximately 16–22 nucleotides, which bind to complementary sequences (seed sequences) in the 3′UTR of target mRNAs and mediate either their decay or translation inhibition [21, 22]. miRNAs are encoded within intronic, intergenic regions or in polycistronic clusters [19, 23], and their biogenesis starts with a RNA polymerase II-dependent transcription of a primary transcript (pri-miRNA), which is then maturated by a nuclear microprocessor complex (RNase III Drosha and its mammalian double-stranded RNA-binding partner DGCR8). This leads to the release of a pre-miRNA, which is then exported into the cytoplasm by Exportin-5, where the RNase III Dicer1, together with its binding partner TARP2 (T-cell receptor gamma-chain constant region), removes the pre-miRNA hairpin loop and generates a miRNA duplex of mature miRNA (guide strand) and of a complementary strand (passenger strand or miRNA^*∗*^). The guide strand and the miRNA^*∗*^ are then associated with Argonaute proteins and incorporated into the RNA-induced silencing complex (RISC). A second maturation step is initiated within the RISC to separate both strands and the mature miRNA binds to the 3′UTR of target mRNAs. Recent evidence also indicates a pathophysiological role of the passenger strand of miRNA (miRNA^*∗*^) in specific conditions, although it frequently appears to be degraded and devoid of any functions [19, 21, 23, 24].

**Figure 1:**
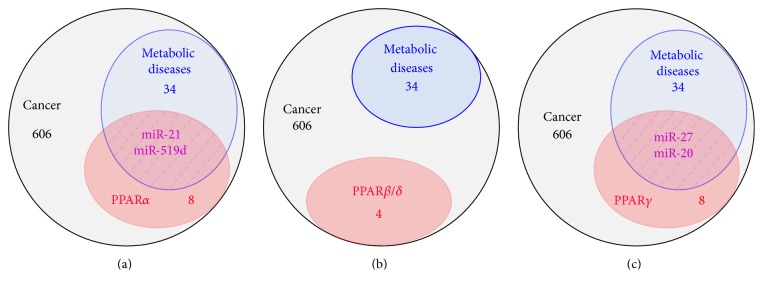
Human miRNAs targeting PPAR isoforms and involved in metabolic diseases and cancer. MetaCore pathway analysis software from Thomson Reuters was used to identify experimentally the number of validated human miRNAs involved in cancer (grey circle). Among those, the numbers of miRNAs involved in metabolic diseases, also identified by MetaCore pathway analysis, are indicated in blue circles. In red circles are the number of miRNAs identified using miRWalk 2.0 atlas and targeting PPAR*α* (Panel (a)), PPAR*β*/*δ* (Panel (b)), and PPAR*γ* (Panel (c)). The identities of miRNAs targeting specific PPAR isoforms and involved in both cancer and metabolic diseases are indicated in violet. miRWalk 2.0 atlas is a software integrating 12 different prediction algorithms (miRWalk 2.0, MicroT4, miRanda, miRBridge, miRDB, miRMap, miRNAMap, PICTAR2, PITA, RNA22, RNAhybrid, and TargetScan) for identification of miRNAs target mRNAs.

More than 2000 miRNAs have been identified and it is considered that 60% of human genes are regulated by miRNAs with around 45 000 miRNA targets within the transcriptome [21–23, 25]. miRNAs act within an intricate regulatory network, where one specific miRNA can control the expression of several hundred mRNAs and conversely one mRNA can be targeted by several miRNAs [19, 23]. Through their wide action, miRNAs are involved in the control of almost all cellular functions and alterations of their expression/activity are observed in various pathological conditions including metabolic diseases and associated cancers [2, 21, 22, 25–29]. The bulk of the studies investigating PPARs and associated cofactors/repressors (e.g., RXR, NCOR) regulation by miRNAs has been performed in the frame of metabolic diseases, where the role of PPARs is the best characterized. Indeed, bioinformatics analyses using the miRWalk 2.0 platform (http://zmf.umm.uni-heidelberg.de/apps/zmf/mirwalk2/index.html), which integrates different prediction software programs for miRNA-mRNA interactions, point to multiple candidate miRNAs potentially targeting directly PPAR isoforms. However, only a restricted number of these candidates have been validated by experimental approaches (see Table 1). Combining MetaCore™ and miRWalk 2.0 based analyses in human studies exclusively revealed that 606 miRNAs were implicated in human cancers, and among those 34 are in metabolic disorders (e.g., obesity, diabetes, hepatomegaly, fatty liver diseases, hypertension, dyslipidemia, and other diabetic complications). Among the 606 cancer-related miRNAs, eight were targeting PPAR*α*, four were targeting PPAR*β*/*δ*, and eight were targeting PPAR*γ*. Interestingly, two miRNAs targeting PPAR*α* (miR-21 and miR-519d) and two miRNAs targeting PPAR*γ* (miR-27 and miR-20) were also previously associated with metabolic diseases (Figure 1). Although such predictive analyses using available software programs are subject to multiple biases and should be considered with extreme caution, they suggest that fine-tuning of PPARs signaling by miRNAs may sit at the crossroad between metabolic diseases and cancers in human.

## 3. miRNAs-Dependent Regulation of PPARs in Metabolic Diseases and Cancer

### 3.1. miRNAs-Dependent Regulation of PPAR*α*

**Figure 2:**
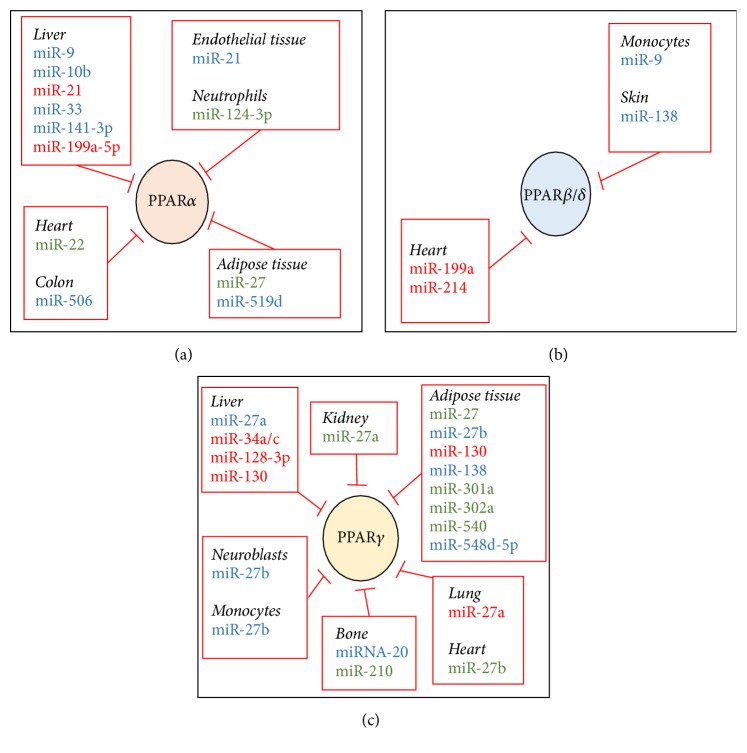
miRNAs targeting PPAR isoforms in specific tissues. miRNAs (also referred to in Table 1) that have been experimentally demonstrated to specifically target PPAR*α* (Panel (a)), PPAR*β*/*δ* (Panel (b)), and PPAR*γ* (Panel (c)) in different tissues are illustrated. miRNAs identified in human studies are in blue, those identified in mouse/rat studies are in green, and those identified in both human and rodents studies are in red.

PPAR*α* is a nutritional sensor adapting metabolic homeostasis to energy deprivation [3]. It is mostly expressed in the liver, where it regulates lipid catabolism (i.e., *β*-oxidation) and critical genes (e.g., fatty acid transport protein 1, CD36, Acyl-CoA oxidase 1, and Carnitine palmitoyltransferase 1) involved in fatty acid transport [68] and in ketogenesis (e.g., Hmgcs2, Hmgcl) [68]. PPAR*α* exerts also an anti-inflammatory function, as evidenced in mouse models of acute inflammation [68]. This effect results from an attenuation of proinflammatory cytokines (e.g., Il-6, Il-1*β*) expression as well as an upregulation of anti-inflammatory factors such as Il-1ra (Il-1 receptor antagonist) or I*κ*B*α* [68]. PPAR*α* is also expressed in other organs such as adipose tissues, heart, skeletal muscles, and kidneys, where it controls also some aspects of the glucose and lipid homeostasis (i.e., *β*-oxidation) [6, 68, 69]. PPAR*α* is usually activated through the binding of specific ligands, in particular unsaturated fatty acids (*ω*-3 fatty acids), eicosanoid derivatives (e.g., 8-hydroxy-eicosatetraenoic acid, prostacyclin), or metabolized fatty acids (e.g., oxidized fatty acids) [6]. Alterations of PPAR*α* expression or activity were associated with a variety of human pathologies such as obesity, liver diseases, inflammation, and cancers [3, 68, 69]. It is now clear that deregulations of specific miRNA can significantly contribute to PPAR*α* abnormal signaling in these pathophysiological conditions (see experimentally validated miRNAs targeting PPAR*α* in Table 1 and Figure 2). Such alterations have been investigated only in specific tissues, such as the liver or adipose tissue, as well as in inflammatory cells and cartilage and specific tumors (e.g., in the colon). Whether PPAR*α* expression/activity is affected by miRNAs in other metabolically active tissues, for example, skeletal muscles or pancreas, remains to be established.

#### 3.1.1. miRNAs-Dependent Regulation of PPAR*α* in the Liver

In the liver, PPAR*α* is implicated in the lipid catabolism and inflammatory processes [68]. miRNAs-dependent alterations of PPAR*α* signaling are reported by numerous studies to contribute to the onset of liver diseases such as nonalcoholic fatty liver disease (NAFLD) [19, 21, 29], chronic diseases associated with viral infections (HBV, HCV) [70], or hepatic cancers [30, 71].

*Hepatic Steatosis.* Two miRNAs were shown to alter PPAR*α* expression in hepatocytes and to lead to steatosis development (Table 1) [19, 69]. Upregulation of miR-199a-5p was observed in various in vivo mouse models of obesity (ob/ob and db/db mice, mice fed a high-fat diet), as well as in liver samples from patients with NAFLD. In vitro analyses of hepatoma cell lines (HepG2 and murine AML12 cells) exposed to fatty acids as a surrogate model of steatosis further confirm an upregulation of miR-199a-5p, which in turn downregulates PPAR*α* and caveolin-1 thereby promoting abnormal cellular redox equilibrium and fatty acids intracellular accumulation [37]. In human hepatic LO2 cells, Zheng et al. uncover another miRNA, miR-10b, upregulated following exposure to fatty acids and having a unique binding site in the PPAR*α* 3′UTR sequence [32]. However, the relevance of miR-10b alterations in human liver metabolic disorders was not evaluated. 

*Hepatic Inflammation and Fibrosis.* PPAR*α* downregulation by miRNAs was recently suggested to trigger hepatic inflammation and fibrosis. Indeed, Loyer et al. [34] reported an upregulation of miR-21 in biliary and inflammatory cells of mice and patients with nonalcoholic steatohepatitis (NASH). They further discover that miR-21 was promoting hepatic inflammation and fibrosis by suppressing PPAR*α* expression in these cells. Interestingly, in mice knockout, specifically for miR-21 in hepatocytes, PPAR*α* expression was not altered, even when mice were challenged with an obesogenic diet, therefore suggesting that, in different cell types, miR-21 may have different activities and/or cellular targets [34, 72]. In hepatic stellate cells (HSCs), which are the main nonparenchymal liver cells contributing to the abnormal extracellular matrix deposition in liver fibrosis, miR-33 and miR-27a/-27b were also found upregulated and to target PPAR*α* and the PPAR*α* cofactor RXR, respectively. Inhibition of these miRNAs with synthetic nucleotides in rat primary and immortalized human HSCs (LX-2 cells) increased PPAR*α* expression concomitantly with a decreased activation of the cells, thus suggesting a tight link between HSC activation and PPAR*α* expression [35, 73]. 

*Hepatic Carcinogenesis.* The role of PPAR*α* in cancer is still debated but few studies suggested that miRNAs-dependent alterations of PPAR*α* expression/activity are relevant for the development of hepatocellular carcinoma (HCC). In particular, high-throughput screening of human HCC samples revealed 28 miRNAs differentially expressed with top hits for miR-9, miR-21, and miR-224 [30]. In addition to miR-21, which is discussed in the previous section, prediction software programs identified conserved miR-9 binding sites within the 3′UTR of PPAR*α*. miR-9 upregulation correlated with tumor invasiveness, cell growth, and tumor stage, but whether this was related to a decreased PPAR*α* expression remains unclear. Indeed, whereas the direct regulation of PPAR*α* by miR-9 in human HCC cells was confirmed by luciferase reporter assay [31], molecular analysis of human Snu-449 and HepG2 cancer cell lines indicated an indirect role for miR-9 overexpression in PPAR*α* downregulation [30].

#### 3.1.2. miRNAs-Dependent Regulation of PPAR*α* in the Adipose Tissue

PPAR*α* assumes important functions in brown adipose tissue and adaptive thermogenesis and browning of white adipose tissue [41]. Although experimental evidence in human showing miRNAs-dependent PPAR*α* regulation in brown/white adipose tissue is scarce, several animal models have suggested such regulatory mechanisms. For example, miR-27a and miR-27b, which are downregulated in mouse brown/white adipose tissue after cold exposure, directly modulate components of the adipocyte transcriptional network including PPAR*α* [41]. MiR-519d, which is increased in subcutaneous white adipose tissue of obese subjects as compared to nonobese individuals, decreases fatty acid catabolism, and increases intracellular lipid accumulation by directly repressing PPAR*α* [39]. Finally, other miRNAs upregulated in brown adipose tissue and/or white adipose tissue of diet-induced obese mice, or during human white and beige adipose differentiation, for example, miR-106b/miR-93 [74, 75] and miR-26a and miR-26b [50], have been correlated with alterations of PPAR*α* expression. However, whether PPAR*α* is a direct target of these miRNAs was not investigated.

#### 3.1.3. miRNAs-Dependent Regulation of PPAR*α* in Other Cell Types/Organs

*Inflammatory Cells and Cartilage.* Functional miRNAs-dependent PPAR*α* alteration in inflammatory processes was suggested by two studies. First, miR-21, which is upregulated in cultured human endothelial cells from umbilical vein exposed to oscillatory shear stress, was shown to directly inhibit PPAR*α* translation [33]. The decreased expression of PPAR*α* in turn promotes AP1-dependent upregulation of VCAM-1 (vascular cell adhesion molecule-1) and MCP-1 (monocyte chemotactic protein-1), which favor the adhesion of inflammatory cells [33]. In a second study, bioinformatics and molecular analyses combined with clinical data identified an increased expression of miR-22 in osteoarthritic cartilage, which was correlated with PPAR*α* downregulation and an increased body-mass-index (BMI) of patients [76]. However, this study did not provide any direct molecular link between miR-22 upregulation and PPAR*α* downregulation.

*Nonhepatic Cancers.* The only evidence that PPAR*α* may potentially behave as a tumor suppressor downregulated by aberrantly expressed miRNAs in transformed cells comes from a study performed in a drug-resistant colon cancer cell line (SW1116) showing that miR-506 overexpression in this cancer cell model directly affects PPAR*α* expression [38]. In another study, the growth inhibitory properties of 1.25-dihydroxyvitamin D3 in human prostate adenocarcinoma cells (LNCaP) were associated with an increased expression of the miR-17/92 cluster, which correlated with PPAR*α* downregulation, but whether miR-17/92 directly target PPAR*α* was not investigated [77].

### 3.2. miRNAs-Dependent Regulation of PPAR*β*/*δ*

PPAR*β*/*δ* is ubiquitously expressed with the highest levels in liver, intestine, kidneys, and skeletal muscles [6, 78]. Major PPAR*β*/*δ* activators are natural ligands such as polyunsaturated fatty acids, prostaglandin derivatives (e.g., prostacyclin), or components of VLDL (Very Low Density Lipoproteins) particles [6]. This PPAR isoform regulates multiple cellular processes including developmental aspects, the lipid metabolism, insulin sensitivity, vascular function, and anti-inflammatory responses [4, 6, 44, 79]. The best-characterized role of PPAR*β*/*δ* has been described in metabolically active tissues. In the liver, PPAR*β*/*δ* appears to increase glucose utilization through the pentose-phosphate pathway and to promote lipogenesis [80]. However, in mice fed an obesogenic diet, activation of PPAR*β*/*δ* surprisingly prevents the development of steatosis [81]. In muscles and white adipose tissue, PPAR*β*/*δ* exerts an adaptive response to fasting and exercise by favoring fatty acids oxidation [82], through the direct induction of key genes involved in this process (e.g., mitochondrial CPT-1 (Carnitine palmitoyltransferase-1) and FoxO1 (Forkhead box protein O1)) [82, 83]. In brown adipose tissue, PPAR*β*/*δ* contributes to adaptive thermogenesis by inducing the expression of UCP-1 and UCP-3 [81, 82] and to *β*-oxidation, by upregulating several genes involved in this process (e.g., long chain acyl-CoA synthetase, Acyl-CoA oxidase). In addition to these well established roles of PPAR*β*/*δ*, this isoform was further implicated in the regulation of multiple other cellular processes including developmental aspects, vascular function, and anti-inflammatory responses [4, 6, 44, 79]. Finally, in cancer, the role of PPAR*β*/*δ* is controversial with evidence pointing at PPAR*β*/*δ* as an oncogene (e.g., in breast and prostate tumors) [84] or as a tumor suppressor (e.g., in colon cancer) [4, 6, 85]. Despite the key functions of PPAR*β*/*δ*, solid experimental evidence indicating miRNAs-dependent regulation of this isoform is very limited and restricted to studies described below (see Table 1 and Figure 2).

#### 3.2.1. miRNAs-Dependent Regulation of PPAR*β*/*δ* in the Liver

Based on Affymetrix microarrays, in vivo inhibition of miR-122 by antisense oligonucleotides (ASO) in mice affected hundreds of hepatic mRNAs including PPAR*β*/*δ* [86]. Whether miR-122 directly targets PPAR*β*/*δ* is still undetermined; however its downregulation following injection of miR-122 inhibitors in mice was suggested to affect circadian clock-dependent energy homeostasis and in particular regulation of lipid transport and catabolism [86].

#### 3.2.2. miRNAs-Dependent Regulation of PPAR*β*/*δ* in the Heart

By stimulating fatty acid utilization in the myocardium, PPAR*β*/*δ* exerts a protective vascular function. In a mouse model of heart failure, an impaired fatty acid oxidation and a metabolic switch towards glycolysis were attributed to the direct repression of PPAR*β*/*δ* by two miRNAs, miR-199a and miR-214, which are upregulated following aortic pressure overload and subsequent heart failure [43].

#### 3.2.3. miRNAs-Dependent Regulation of PPAR*β*/*δ* in Monocytes/Macrophages

PPAR*β*/*δ* exerts an anti-inflammatory function, by promoting the switch from proinflammatory M1 macrophages to the anti-inflammatory M2 macrophages in the liver and in adipose tissue [44]. Bioinformatics and luciferase reporter assays revealed the presence of a functional miR-9 binding site within PPAR*β*/*δ* 3′UTR in human monocytes. Downregulation of PPAR*β*/*δ* and its targets genes was further observed in proinflammatory M1 macrophages treated with lipopolysaccharide (LPS) and correlated with an upregulation of miR-9 in these cells, thus suggesting a potential functional regulation of PPAR*β*/*δ* by miR-9 in monocytes and macrophages [44].

#### 3.2.4. miRNAs-Dependent Regulation of PPAR*β*/*δ* in Other Cell Types/Organs

MiRNAs-dependent PPAR*β*/*δ* regulation was finally reported in hypertrophic scar formation, where PPAR*β*/*δ* promotes proliferation of fibroblasts. A decrease in miR-138 expression was noted in scar tissue as compared to paired normal skin tissues and inversely correlated with the level of PPAR*β*/*δ*. Further analyses using luciferase reporter assays and synthetic miR-138 mimics and inhibitor nucleotides in human hypertrophic scar fibroblasts (hHSFs) confirmed that PPAR*β*/*δ* is a direct target of miR-138 and the functional relevance of this regulatory mechanism in hHSFs proliferation [45].

### 3.3. miRNAs-Dependent Regulation of PPAR*γ*

There are two PPAR*γ* isoforms (PPAR*γ*1 and PPAR*γ*2). PPAR*γ*1 is broadly expressed in adipose tissue, liver, intestine, kidneys, small intestine, immune cells, and endothelium, while PPAR*γ*2 is predominantly expressed in the adipose tissue [2, 3, 6]. Activation of PPAR*γ* is induced mostly by unsaturated fatty acids and endogenous arachidonic acid-derived metabolites (e.g., leukotriene B4 and eicosatetraenoic acid). The best-described functions of PPAR*γ* are to transcriptionally promote adipocyte differentiation and lipogenesis as well as* de novo* lipogenesis in the liver [5, 87]. In addition, PPAR*γ* controls also the expression of various adipocyte genes involved in glucose homeostasis (e.g., Glut4 expression) and endocrine signaling (e.g., adiponectin, resistin, and TNF*α*) affecting insulin sensitivity in other peripheral organs such as liver and muscles [2, 3, 5, 12]. Finally, several other cellular processes including cholesterol transport, kidney function, food intake, and inflammation have been suggested to be modulated by PPAR*γ* isoforms [2, 3, 5, 12]. Consistent with the role of PPAR*γ* in glucose and lipid homeostasis, an abnormal activity of PPAR*γ* is often associated with the development of metabolic disorders (e.g., obesity, type 2 diabetes, and fatty liver disease) [5]. In contrast, in cancer, increasing evidence indicates a beneficial tumor suppressive role for PPAR*γ* (e.g., gastric, pancreatic, and hepatic cancers) [2, 6, 88]. As illustrated in Table 1 and Figure 2, posttranscriptional regulation of PPAR*γ* by miRNAs has been reported in many pathophysiological situations.

#### 3.3.1. miRNAs-Dependent Regulation of PPAR*γ* in the Liver

*Hepatic Fibrosis.* PPAR*γ* is a negative regulator of hepatic stellate cells (HSCs) activation [89]. The induction of various miRNAs expressed in nonalcoholic steatohepatitis (NASH) and fibrosis correlated with PPAR*γ* downregulation and overexpression of profibrogenic markers like *α*-SMA. Among those miRNAs, upregulation of miR-34a/-34c [52], miR-128-3p [53], and miR-130a/miR-130b [63] in activated human or rat HSCs was reported to directly bind the 3′UTR of PPAR*γ* and to repress its expression. miRNAs-dependent PPAR*γ* downregulation in hepatic fibrosis can also occur through indirect mechanisms. For example, in HSCs from mice treated with CCL4 to induce fibrosis, miR-132 is downregulated thereby leading to an increase of one of its targets, MeCP2 (Methyl CpG binding protein 2), and repression of PPAR*γ* transcription* via* different epigenetic mechanisms [90]. 

*Hepatitis C Virus (HCV) Infection.* Infection of Huh-7.5 hepatoma cells with a HCV-derived JFH1 strain induces expression of miR-27a. This miRNA directly targets PPAR*γ* thereby reducing lipid synthesis and increasing lipid secretion [91], two processes likely promoting HCV replication and virions egress. 

*Hepatic Carcinogenesis.* PPAR*γ* has a tumor suppressive function in hepatocarcinogenesis [7, 51, 92–95]. PPAR*γ* downregulation in HCC correlated with upregulation of specific miRNAs [3, 93, 96], among which the best characterized ones are miR-130b and miR-27a. These two miRNAs directly target PPAR*γ* and decrease its expression thus promoting cancer cells growth and aggressiveness [47, 55]. 

#### 3.3.2. miRNAs-Dependent Regulation of PPAR*γ* in Adipose Tissue

*Adipocyte Differentiation.* Regulation of PPAR*γ* activity/expression by miRNAs represents an important posttranscriptional mechanism controlling adipocyte differentiation. Several miRNAs in murine and human preadipocytes, including miR-540, miR-302a, miR-138, miR-548d-5p, miR-130, and miR-27, were described to bind the 3′UTR of PPAR*γ* and to decrease its expression thus preventing differentiation towards mature adipocytes [54, 57–59, 67, 97, 98]. In particular, miR-130 was reported to be downregulated in mice fed an obesogenic diet and in adipocytes of obese and type 2 diabetic patients, who also have high levels of PPAR*γ* in adipose tissues and a low abundance of preadipocytes [54, 56, 62]. Further in vitro analyses using embryonic fibroblasts-derived preadipocytes (3T3-L1) indicated that synthetic nucleotides mimicking or inhibiting miR-130a were able to modulate PPAR*γ* expression and its downstream target genes involved in glucose and lipid metabolism [56]. miR-27a and miR-27b are other key miRNAs regulating adipocyte differentiation, and both are downregulated during adipocyte differentiation, thus leading to an induction of PPAR*γ* [59]. Consistent with this role, expression of miR-27a/-27b is lower in obese ob/ob mice as compared to lean animals and decreases during adipogenic differentiation of 3T3-L1 cells and mouse bone marrow derived mesenchymal stem cells (OP9 cell line). In the same study, miR-27a/miR-27b mimic nucleotides decreased PPAR*γ* expression and prevented adipocyte differentiation. However, experimental evidence indicated that the mechanisms by which miR-27 affect PPAR*γ* expression are indirect [98]. 

*Inflammation.* The role of PPAR*γ* in adipose tissue inflammation is still poorly characterized, but downregulation of miR-301a, which directly targets PPAR*γ*, was correlated with the production of proinflammatory cytokines in obese mice and in 3T3L1 preadipocytes [65].

#### 3.3.3. miRNAs-Dependent Regulation of PPAR*γ* in Bone Marrow

The commitment of mesenchymal stem cells (MSCs) in the bone marrow towards osteogenic or adipogenic differentiation might be also tightly dependent on PPAR*γ* regulation by miRNAs. Indeed, miR-548d-5p, which is downregulated during adipogenic differentiation of human bone marrow derived MSCs, targets the 3′UTR of PPAR*γ*. Overexpression of this miRNA abrogates adipogenic differentiation and increases the osteogenic potential of MSCs by downregulating PPAR*γ* and C/EBP*α* [58]. Induction of other miRNAs such as miR-20 during osteogenic differentiation leads also to a direct downregulation of PPAR*γ* [46]. In addition, miR-17-5p and miR-106a also promote adipogenesis and inhibit osteogenic differentiation in human adipose derived MSCs by indirect mechanisms, which increase C/EBP*α* and PPAR*γ* expressions [99]. Finally, alterations of the osteogenic/adipogenic differentiation balance is an important component of specific osteogenic-related disorders such as osteoporosis and deregulation of the expression of miRNAs targeting PPAR*γ*, for example, miR-210, have been involved in these diseases [64, 100].

#### 3.3.4. miRNAs-Dependent Regulation of PPAR*γ* in the Heart

Upregulation of miR-27b expression was shown in heart-specific smad4 knockout mice, which develop cardiac hypertrophy [61]. Overexpression of this miRNA specifically in cardiomyocytes using transgenic mice was sufficient to induce cardiac hypertrophy through PPAR*γ* downregulation [61]. Conversely, treatment of a mouse model of heart failure with miR-27b inhibitors (antagomirs) improved cardiovascular functions by increasing PPAR*γ* expression [61]. Similarly, in vivo inhibition of miR-128 by antagomirs protected cardiomyocytes from apoptosis in a model of myocardial ischemia/reperfusion injury and increased PPAR*γ* expression in neonatal rat ventricular myocytes (NRVM) [101]. However, whether miR-128 modulates PPAR*γ* through direct or indirect mechanisms was not assessed in this study.

#### 3.3.5. miRNAs-Dependent Regulation of PPAR*γ* in Other Cell Types/Organs

In addition to its role in hepatocytes, adipocytes, and cardiomyocytes, the relevance of miR-27b targeting of PPAR*γ* was also highlighted in several other tissues (inflammatory cells, renal tubular cells, and pulmonary endothelial cells as well as neuroblastoma).

*Inflammatory Cells.* PPAR*γ* is a potent inhibitor of M1 macrophage activation (Th1 proinflammatory macrophages) and promoter of M2 macrophage activation (Th2 anti-inflammatory macrophages) [102]. Upregulation of miR-27b in human macrophages upon LPS exposure was demonstrated to directly target PPAR*γ* and to elicit a Th1 differentiation [49]. Although these findings suggest that miR-27b-dependent downregulation of PPAR*γ* may represent a key process in macrophage polarization, whether miR-27b controls M2 macrophage activation* via* PPAR*γ* was however not investigated. 

*Kidneys.* Upregulation of miR-27a occurs in glucose-stimulated rat renal proximal tubular cell line (NRK-52E cells) and renal tubular epithelial cells of streptozotocin-induced diabetic rats. In these cellular contexts, the increased miR-27a expression was shown to trigger PPAR*γ* downregulation, which in turn promoted renal fibrosis [60]. 

*Lung.* In mice and human pulmonary artery endothelial cells (HPAECs), hypoxia upregulates miR-27a expression and decreases PPAR*γ* expression [103]. Given the important antiproliferative, and antithrombotic and vasodilatory effects of PPAR*γ* on the lung vasculature [104], upregulation of miR-27a may thus represent an important contributor to the development of pulmonary hypertension. 

*Neuroblastoma Cells.* Although miR-27b-dependent downregulation of PPAR*γ* promotes cell proliferation in HCC, it may lead to opposite effects in other cancers [7, 51, 92–95]. This is the case in the SK-N-AS neuroblastoma cells and derived mouse xenografts, where miR-27b was shown to repress PPAR*γ* expression resulting in a decreased inflammatory response and tumor growth [51, 105, 106].

## 4. Indirect Regulation of PPARs by miRNAs

**Figure 3:**
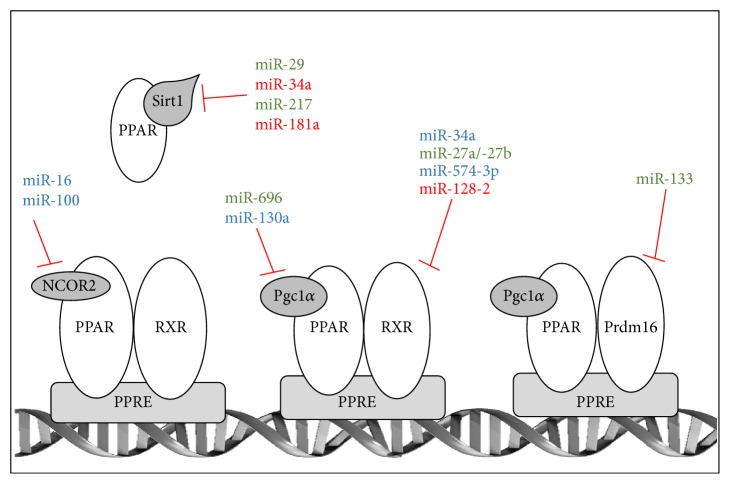
miRNAs targeting PPAR transcriptional partners, cofactors/repressors, and other regulators. miRNAs that have been experimentally demonstrated to specifically target PPAR transcriptional partners (RXR and Prdm16), PPAR cofactors (Pgc1*α*), PPAR repressors (NCOR2), and other PPAR regulators (Sirt1) are illustrated. miRNAs identified in human studies are in blue, those identified in mouse/rat studies are in green, and those identified in both human and rodents studies are in red.* PPAR: peroxisome proliferator-activated receptor α*, *β*/*δ*, or *γ*;* RXR: Retinoid-X-Receptor; Prdm16: PR domain-containing 16; Sirt1: Sirtuin-1; NCOR2: nuclear receptor corepressor 2 (SMRT); Pgc1α: peroxisome proliferator-activated receptor gamma coactivator 1; PPRE: peroxisome proliferator response element*.

The activity of PPARs is tightly linked to the binding of transcriptional partners (i.e., RXR, Prdm16), cofactors/repressors (e.g., PGC1*α*, NCOR), or other regulators (e.g., Sirt1) [3]. Most of the PPARs binding partners and cofactors are also finely tuned by specific miRNAs, which thereby indirectly regulate the expression/activity of PPARs isoforms [18, 107–112]. A brief overview of miRNAs targeting PPARs binding partners and cofactors is provided in the next section (see Figure 3).

### 4.1. miRNAs-Dependent Regulation of PPARs Binding/Heterodimerization Partners

#### 4.1.1. miRNAs-Dependent Regulation of RXRs

RXR isoforms (RXR*α*/*β*/*γ*) are the obligate binding partners for PPARs. Together they form heterodimeric complexes and induce gene transactivation by binding to PPAR response elements (PPREs) [3]. As illustrated in Figure 3, several miRNAs have been reported to directly target RXR isoforms thus affecting indirectly PPARs activities [108, 109, 113, 114]. For example, miR-128-2 was shown to suppress cholesterol efflux in HepG2 cells and in the liver of diet-induced obese mice by binding to the 3′UTR of RXR*α* and of ATP-binding cassette transporters (ABCA1 and ABCG1) and repressing their expressions [114]. Chondrogenesis, which is inhibited by RXR*α*, was also promoted in mesenchymal stem cells by miR-574-3p, which downregulates specifically RXR*α* expression [113]. Interestingly, specific miRNAs targeting PPAR*γ*, that is, miR-34a and miR-27a/b, also control RXR*α* expression in liver cells [73, 98, 109]. Indeed upregulation of miR-34a, which was correlated with fibrosis development, downregulates RXR*α* by binding within the coding region and not the 3′UTR of this isoform in hepatocytes [109]. In the case of miR-27a and miR-27b, these two miRNAs were upregulated in rat activated HSCs and decrease RXR*α* expression through 3′UTR-dependent mechanisms [73, 98]. It thus appears that abnormal miRNAs-dependent inhibition of RXR*α* in distinct liver cells contributes to the development of hepatic fibrogenesis. Finally, inhibition of RXR*α* by upregulation of miR-27a was also reported in aggressive rhabdomyosarcoma (RMS) [108]. Altogether, these studies suggest that particular miRNAs, such as miR-34a and the miR-27 family, may affect PPARs signaling by simultaneously targeting different key players in this pathway.

#### 4.1.2. miRNAs-Dependent Regulation of Prdm16

During brown adipogenesis, Prdm16 (PR domain-containing 16) instead of RXR*α* heterodimerizes with PPAR*γ*2 and mediates brown adipocyte differentiation [115]. MiR-133a was demonstrated to regulate directly Prdm16 expression in immortalized brown preadipocytes [18] and inhibition of miR-133a and miR-133b led to an increased expression of adipogenic markers including PPAR*γ* as well as differentiation towards mature brown adipocytes [18, 116, 117].

#### 4.1.3. miRNAs-Dependent Regulation of PGC1*α*

PGC1*α* is a critical transcriptional coactivator of PPAR*γ* in brown preadipocytes and of PPAR*α* in white preadipocytes (3T3-L1 cells) [118]. To date only two miRNAs have been described in hepatocytes to directly target PGC1*α* mRNA: (i) miR-696, which is upregulated with obesity, decreases PGC1*α* expression in the liver of ob/ob mice [119] and (ii) miR-130a, which is downregulated in HBV-infected human hepatocytes, increases PGC1*α* and PPAR*γ* expression thus favoring HBV replication [120].

#### 4.1.4. miRNAs-Dependent Regulation of NCOR

In the absence of PPARs ligands, the transcriptional activity of PPARs is inhibited by the binding of corepressors such NCOR proteins [12]. miRNAs-dependent regulation of NCOR proteins is supported by two studies showing that (i) miR-16 in LPS-activated human monocytes (U937) and biliary epithelial cells (H69) targets SMRT (NCOR2), which leads to NF-*κ*B-mediated transactivation of the IL-8 gene [107], and (ii) miR-100 targets SMRT (NCOR2) in glioblastoma cells thereby inhibiting their proliferation [112].

#### 4.1.5. miRNAs-Dependent Regulation of Sirtuin-1

The NAD-dependent deacetylase Sirtuin-1 (Sirt1) is a critical regulator of PPAR signaling and of energy homeostasis [121]. Posttranscriptional control of SIRT1 and other sirtuins by miRNAs represents important regulatory mechanism for this protein family and has been extensively reviewed elsewhere [111]. Among the various miRNAs directly targeting SIRT1, miR-217 [122], miR-181a [123], miR-29 [124], and miR-34a [125] in particular were shown to affect hepatic lipid metabolism, insulin sensitivity, or carcinogenesis by modulating SIRT1 expression. Of note, miR-34a is also a direct regulator of PPAR*γ* and RXR*α* expression [111, 125–128] therefore supporting again the biological relevance of fine-tuning PPARs signaling by modulating several factors involved in this transcriptional pathway.

## 5. miRNAs Regulated by PPARs

**Figure 4:**
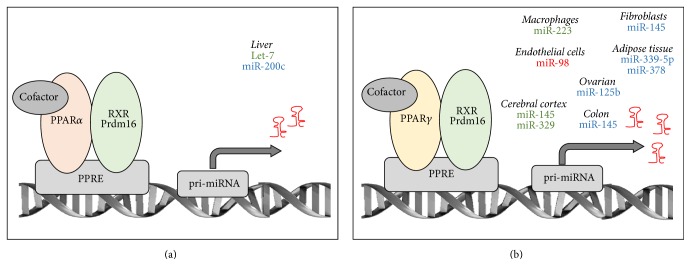
miRNAs expression induced by PPAR*α* and PPAR*γ*. Induction of miRNAs by PPAR*α* (Panel (a)) and PPAR*γ* (Panel (b)) binding to PPRE in pri-miRNA promoters in specific tissues is indicated. miRNAs identified in human studies are in blue, those identified in mouse/rat studies are in green, and those identified in both human and rodents studies are in red.* RXR: Retinoid-X-Receptor; Prdm16: PR domain-containing 16; PPRE: peroxisome proliferator response element*.

Recent evidence indicates that the expression of particular miRNAs can also be under the transcriptional control of PPARs [129] (see Figure 4). Most of the studies reviewed here rely on the identification of PPAR response elements (PPREs) within the promoter of genes encoding pri-miRNAs or on the effects of PPARs agonists [17, 96, 130, 131]. miRNAs described to date to be regulated by PPARs are short-listed in Table 2.

### 5.1. PPAR*α*- and PPAR*β*/*δ*-Dependent Regulation of miRNAs Expression

Limited information is available on PPAR*α*- and PPAR*β*/*δ*-dependent regulation of miRNAs expression. PPAR*α* was suggested to promote the expression of let-7 and miR-200c in hepatic cancer cells. Indeed, expression of let-7, which targets c-myc in hepatocytes, was decreased in mice treated with the PPAR*α* agonist Wy-14,643, which in turn fostered myc-dependent liver oncogenesis [71]. In Huh-7 hepatoma cells, PPAR*α* in synergy with another nuclear receptor, that is, LRH-1 (liver receptor homolog-1), was proposed also to drive miR-200c transcription through a direct binding to its promoter [132]. Although the role of miR-200c in HCC was not investigated in this study, miR-200 was previously shown to have tumor suppressive activities by inhibiting cell migration [135]. Regarding PPAR*β*/*δ*, treatment of HUVEC endothelial cells with a PPAR*β*/*δ* agonist (GW501516) led to an increase of miR-100 expression, which improved lipidemia and vascular function [136]. However, as for the study using PPAR*α* agonists, a direct binding of PPAR*β*/*δ* to the miR-100 promoter was not investigated and additional experiments are required to confirm these data and exclude off-target effects of pharmacological agonists of PPAR*β*/*δ*.

### 5.2. PPAR*γ*-Dependent Regulation of miRNAs Expression

In contrast to the other PPAR isoforms, PPAR*γ* was reported to regulate several miRNAs in distinct pathophysiological processes (see Table 2). 

*Endothelial Functions.* miR-98, which is reduced in endothelial cells of patients suffering from idiopathic pulmonary hypertension (IPAH) and of mouse models of this disease, directly targets endothelin-1 (ET1). PPAR*γ* was shown to exert a beneficial role in pulmonary hypertension (PH) by attenuating, likely through activation of miR-98, ET1 expression. In support of this hypothesis, activation of PPAR*γ* with specific agonists (e.g., rosiglitazone) restores miR-98 expression in hypoxic mouse and in primary human pulmonary artery endothelial cells (PAECs); however whether PPAR*γ* is a direct regulator of miR-98 was not assessed [103]. 

*Adipocytes Differentiation and Function.* PPAR*γ* agonists (rosiglitazone and pioglitazone) modulated the expression of 27 different miRNAs in human subcutaneous and visceral adipocytes. Among those, miR-329, miR-145, and miR-339-5p are involved, based on predictive bioinformatics analyses, in metabolic (e.g., insulin signaling) and proliferative (e.g., Wnt/*β*-catenin signaling) pathways [17, 134]. Interestingly, miR-329 and miR-145 contain a PPRE in their promoters and both miRNAs also bind to PPAR*γ* 3′UTR, thus suggesting the existence of positive feedback loop mechanisms regulating expressions of these miRNAs and PPAR*γ* [17]. In human subcutaneous adipocytes and bovine preadipocytes, PPAR*γ* also induces the expression of miR-378, which is located in the first intron of PPAR*γ* coactivator-1*β* (PGC1*β*) [98, 134, 137]. Finally, a list of potential miRNAs, directly regulated by PPAR*γ* and involved in 3T3-L1 adipose differentiation, was identified by crossing datasets of miRNAs containing putative PPAR*γ* binding site with datasets of miRNAs altered during 3T3-L1 differentiation. Authors of this study identified miR-103-1, miR-182/miR-96/miR-183, miR-205, and miR-378 as potential PPAR*γ*-regulated miRNAs, whose expression was further induced in 3T3-L1 cells treated by rosiglitazone. Chip analyses also revealed that these miRNAs are directly regulated by PPAR*γ* through PPRE present in their host genes (PanK3 and PGC1*β*) [138]. 

*Inflammation.* Exposure of bone marrow derived macrophages (BMDMs) to Th2 stimuli (i.e., IL-4) triggers the expression of miR-223 through a direct binding of PPAR*γ* in PPREs within the promoter of pre-miR-223. This effect was further enhanced by a PPAR*γ* agonist (i.e., pioglitazone) and inhibited by a PPAR*γ* antagonist (i.e., GW9662). Since PPAR*γ*-dependent M2 activation is inhibited in BMDMs from miR-223 knockout mice, these data suggest that miR-223 and its target genes (e.g.,* Rasa1* and* genuine*) are key effectors of macrophages polarization [133]. 

*Fibrosis.* Whether PPAR*γ* may control fibrotic processes through miRNAs-dependent mechanisms is not well established, but one study supports this concept. Indeed, Dharap et al., reported that miR-145, which contains a PPAR response element in its promoter [17, 96], was increased in rosiglitazone-treated hypertrophic scar fibroblasts (HSFDs), thus leading to a direct decrease of SMAD3 expression and collagen synthesis [131]. 

*Carcinogenesis.* A direct effect of PPAR*γ* on the expression of specific miRNAs through binding of PPRE in their promoters was demonstrated for three different types of cancer cell lines. In hepatoma HepG2 cells, miR-122 was strongly induced in cells treated with DNA methylation or histone deacetylase inhibitors* via* a direct binding of PPAR*γ*/RXR*α* in the pre-miR-122 promoter [139]. In ovarian cancer cells (i.e., Ovcar3, CaOv3, and Skov3 cells), PPAR*γ* also directly regulates the transcription of miR-125b and silencing of miR-125 impaired the growth inhibitory capacity of PPAR*γ* agonists [130]. Finally, miR-145, which is downregulated in colorectal cancer cells (Caco2, Sw480, HCT116, and HT29) and colorectal tumor tissues, is induced by the PPAR*γ* agonist (i.e., rosiglitazone)* via* direct binding of PPAR*γ* to PPRE in the promoter encoding pre-miR-145 [96, 131].

## 6. miRNAs-Based Therapies to Target PPARs Expression/Activity

Targeting tissue-specific miRNAs with pharmacological compounds may represent novel and valuable alternative therapeutic approaches to PPAR agonists or antagonists [10–13]. Different methods have been developed to modulate miRNA expressions in vivo. Of particular interest are chemically modified synthetic oligonucleotides inhibiting or mimicking endogenous miRNAs that display increased affinity for their targets and great stability in the serum. Currently, these oligonucleotides bear modifications on the 2′- or 3′-position of the nucleic acid ribose backbone. For example, antagomiRs (3′-cholesterol-conjugated, 2′-O-Me oligonucleotides with terminal phosphorothioate modifications), antisense modified oligonucleotides (AMO) (2′-O-methoxyethyl phosphorothioate modified antisense oligonucleotide or 2′-fluoro-modified antisense oligonucleotides), or locked nucleic acids (LNA) represent potent inhibitors of miRNAs expression/activity. These synthetic nucleotides are usually administered by intravenous injection and hopefully soon orally with a good efficiency [140, 141]. When administered by intravenous injection they can broadly reach every tissue but tends to accumulate in particular organs, such as the liver or the kidneys [26]. Special formulations such as liposomes or polyethylenimine-formulated nanoparticles as miRNA nanocarriers have been developed to improve tissue-specific distribution, circulation time, and clearance of the miRNAs-like compounds [26, 142, 143]. Of particular interest, microvesicles (MVs) were shown to represent efficient and functional miRNA delivery tools as it was demonstrated in animals and in the case of miR-130b [144, 145]. Other alternative methods to target specific tissues have been also developed such as inclusion of oligonucleotides into liposomal or oleic-based nanoparticles, which target preferentially the liver [142]. Finally, viruses with specific tropisms, for example, adenoassociated viruses (AAV), have been used in animal models to robustly express or inhibit specific miRNAs in particular tissues and may represent an interesting alternative to chemically modified nucleotides [26]. Importantly, abnormal levels of circulating miRNAs stimulate toll-like receptors therefore promoting inflammation and favoring the development of chronic diseases such as metabolic and cardiovascular disorders as well as cancers. Interestingly, chemically modified synthetic oligonucleotides, in particular those modified in the 2′ position of the ribose, have the ability to reduce, but not completely prevent, such unintended immune responses [26, 146].

Several miRNA inhibitors have been tested in preclinical studies with rodents or primates in the context of various pathologies (e.g., miR-155 in inflammatory diseases, miR-208 in cardiac remodeling) including metabolic diseases (e.g., miR-103/107 for type 2 diabetes and obesity) [26]. However, only few of them, for example, miR-122 inhibitors (Miravirsen) to treat HCV infection, are currently being tested in human clinical trials [26, 147–149]. Unfortunately, none of the miRNAs known to potentially target PPAR isoforms are under clinical trials in human. Only preclinical studies were performed for miR-33 and miR-21 [26], which targets directly PPAR*α* (Figures 2-3). In African green monkeys, inhibition of mir-33a/b with specific antagomiRs increased the hepatic expression of ABCA1, thus leading to an increase of HDL (high density lipoprotein) and a decrease in VLDL (very low density lipoproteins) and triglycerides plasma levels [150]. Inhibition of miR-21 in mice with an antisense oligonucleotide prevented hepatic lipid accumulation in animals fed an obesogenic diet [151].

The (pre)clinical use of synthetic nucleotides mimicking endogenous miRNAs is less developed compared to miRNAs inhibitors and currently only miR-34 mimics nucleotides are tested to treat some cancers [26, 152]. MRX34, a liposome-formulated miR-34 mimic-based drug is currently in phase I study for melanoma patients. This miR-34 mimic achieved positive outcomes as a monotherapeutic agent in patients with renal cell carcinoma, acral melanoma, and HCC (http://www.mirnatherapeutics.com/pipeline/mirna-pipeline.html) [26]. However, other in vivo studies indicated that miR-34a/c could also activate hepatic stellate cells and promote fibrogenesis by targeting PPARy and repressing RXR*α* and Sirt1 [52, 109]. Other miRNAs, such as miR-27 or miR-9, are also able to regulate the expression/activities of different PPARs isoforms in distinct tissues. Therefore, although miRNAs-based therapies are promising, the potential pleiotropic effects of systemic administration of pharmacological miRNAs inhibitors or mimics call also for cautiousness in their therapeutic use since they can likely lead, as in the case of PPARs agonists/antagonists, to conflicting and unwanted side effects.

## 7. Conclusion

The pivotal role of abnormal PPARs signaling in the development and the progression of various pathologies including metabolic diseases, inflammation, and cancer is now well established. However, the mechanisms and extent to which miRNAs contribute to alterations of PPARs expressions and/or activities in physiopathological conditions are currently still poorly understood and represent an important developing field of research. Conversely, the fact that PPARs can drive the expression of specific miRNAs, which may target in turn hundreds of different mRNAs, opens also a new dimension in our understanding of the physiological and pathological roles of PPARs isoforms. Given the tissue-specific and pleiotropic action of PPARs in various cellular processes described herein, it is likely that posttranscriptional regulation of PPARs and related cofactors by miRNAs is tissue- and process-specific. In addition, the simplistic view that only changes in the intracellular levels of miRNAs impact the expression of target genes is likely incorrect. Indeed increasing evidence indicates that the activity and bioavailability of miRNAs are also key factors to consider in these regulatory mechanisms. This concept is further supported by the emerging role of long noncoding RNAs [31] and RNA-binding proteins, which could interfere with the activity/expression of specific miRNAs [153, 154] and regulation of their target genes. Further studies are thus required to deepen our knowledge of miRNAs-based posttranscriptional regulatory mechanisms controlling PPARs expressions and activities.

## Figures and Tables

**Table 1a:** (a) PPAR*α*

miRNA	Biological process	Cell/tissue	Reference
*Human studies*			

miR-9	Cancer cell invasion and proliferation	HCC tissue Hepatic cell lines	Drakaki et al., 2015 [30]
	Lipid metabolism	HCC tissue Hepatic cell lines	Cui et al., 2015 [31]

miR-10b	Hepatic steatosis	Hepatic cell lines	Zheng et al., 2010 [32]

miR-21	Vascular inflammation	Endothelial cell lines	Zhou et al., 2011 [33]
	Liver cell injury Inflammation Fibrosis	Liver tissue Primary biliary and hepatic inflammatory cells	Loyer et al., 2015 [34]

miR-33	Liver fibrosis	Hepatic stellate cell line	Li et al., 2014 [35]

miR-141-3p	HBV replication	Hepatic cell line	Hu et al., 2012 [36]

miR-199a-5p	Hepatic steatosis	Liver tissue Hepatic cell lines	Li et al., 2014 [37]

miR-506	Drug resistance	Colon cancer cell line	Tong et al., 2011 [38]

miR-519d	Adipocyte differentiation	White adipose tissue Primary preadipocytes	Martinelli et al., 2010 [39]

*Rodent studies*			

miR-21	Liver cell injury Inflammation Fibrosis	Liver tissue Primary biliary and hepatic inflammatory cells	Loyer et al., 2015 [34]

miR-22	Cardiac hypertrophy Cardiac contractility	Heart tissue Primary neonatal cardiomyocytes	Gurha et al., 2013 [40]

miR-27	Adipocyte differentiation	Brown/white adipose tissue, primary adipose derived stromal cells, brown preadipocyte cell line	Sun et al., 2014 [41]

miR-124-3p	Protein secretion	Isolated neutrophils	Baek et al., 2008 [42]

miR-199a-5p	Hepatic steatosis	Liver tissue Hepatic cell lines	Li et al., 2014 [37]

**Table 1b:** (b) PPAR*β*/*δ*

miRNA	Biological process	Cell/tissue	Reference
*Human studies*			

miR-199a miR-214	Mitochondrial metabolism	Myocardium Primary cardiomyocytes	El Azzouzi et al., 2013 [43]

miR-9	Inflammation	Isolated monocytes	Thulin et al., 2013 [44]

miR-138	Wound healing, Proliferation, migration	Skin tissue Hypertrophic scar fibroblasts	Xiao et al., 2015 [45]

*Rodent studies*			

miR-199a miR-214	Mitochondrial metabolism	Myocardium Primary cardiomyocytes	El Azzouzi et al., 2013 [43]

**Table 1c:** (c) PPAR*γ*

miRNA	Biological process	Cell/tissue	Reference
*Human studies*			

miR-20	Osteogenic differentiation	Bone marrow derived stromal cell line	Zhang et al., 2011 [46]

miR-27a	Proliferation	HCC tissue Hepatic cell lines	Li et al., 2015 [47]
	Proliferation	Lung tissue Pulmonary endothelial cell lines	Kang et al., 2013 [48]

miR-27b	Inflammation	Isolated monocytes Monocyte cell line	Jennewein et al., 2010 [49]
	Adipocyte differentiation	Adipose derived stromal cell line	Karbiener et al., 2009 [50]
	Tumor growth and progression	Neuroblastoma cell line	Lee et al., 2012 [51]

miR-34a miR-34c	Liver fibrosis	Primary hepatic stellate cells Hepatic stellate cell line	Li et al., 2015 [52]

miR-128-3p	Liver fibrosis	Primary hepatic stellate cells Hepatic stellate cell line	Povero et al., 2015 [53]

miR-130	Adipocyte differentiation	Primary preadipocytes	Lee et al., 2011 [54]
	Epithelial-mesenchymal transition Cancer cell migration and invasion	HCC tissue Hepatic cell lines	Tu et al., 2014 [55]

miR-130a	Type 2 diabetes mellitus	White adipose tissue Adipocyte cell line	Jiao et al., 2015 [56]

miR-138	Adipocyte differentiation	Primary adipose derived stromal cells	Yang et al., 2011 [57]

miR-548d-5p	Adipocyte differentiation	Bone marrow derived stromal cells	Sun et al., 2014 [58]

*Rodent studies*			

miR-27	Adipocyte differentiation	White adipose tissue, Primary white adipocytes Primary adipose derived stromal cells Preadipocyte cell line	Kim et al., 2010 [59]
	Adipocyte differentiation	Brown/white adipose tissue Primary adipose derived stromal cells Brown pre-adipocyte cell line	Sun et al., 2014 [41]

miR-27a	Proliferation	Lung tissue Pulmonary endothelial cell lines	Kang et al., 2013 [48]
	Renal fibrosis	Kidney tissue Kidney tubular epithelial cells	Hou et al., 2016 [60]

miR-27b	Cardiac hypertrophy Heart failure	Myocardium Primary cardiomyocytes	Wang et al., 2012 [61]

miR-34a miR-34c	Liver fibrosis	Primary hepatic stellate cells Hepatic stellate cell line	Li et al., 2015 [52]

miR-128-3p	Liver fibrosis	Primary hepatic stellate cells Hepatic stellate cell line	Povero et al., 2015 [53]

miR-130	Adipocyte inflammation	Preadipocyte cell line	Kim et al., 2013 [62]
	Liver fibrosis	Primary hepatic stellate cells Hepatic stellate cell line	Lu et al., 2015 [63]

miR-130a	Type 2 diabetes mellitus	White adipose tissue Adipocyte cell line	Jiao et al., 2015 [56]

miR-210	Osteoporosis	Primary bone marrow derived stromal cells	Liu et al., 2015 [64]

miR-301a	Adipocyte inflammation	White adipose tissue Preadipocyte cell line	Li et al., 2016 [65]

miR-302a	Adipocyte differentiation	White adipose tissue Pre-adipocyte cell line	Jeong et al., 2014 [66]

miR-540	Adipocyte differentiation	Primary adipose derived stromal cells	Chen et al., 2015 [67]

**Table 2a:** (a) PPAR*α*

miRNA	Biological process	Organism	Cell/tissue	Reference
Let-7	Proliferation	Mouse	Liver tissue, HCC cell line	Shah et al., 2007 [71]

miR-200c	Migration	Human	HCC cell line	Zhang et al., 2011 [132]

**Table 2b:** (b) PPAR*γ*

miRNA	Biological process	Organism	Cell/tissue	Reference
miR-98	Endothelial dysfunction Pulmonary hypertension	Mouse Human	Primary pulmonary artery endothelial cells	Kang et al., 2016 [103]

miR-125b	Proliferation Apoptosis	Human	Ovarian cancer tissue, Ovarian cancer cell lines	Luo et al., 2015 [130]

miR-145	Inflammation Oxidative stress	Rat	Cerebral cortex, Pheochromocytoma cell line	Dharap et al., 2015 [17]
	Collagen synthesis	Human	Hypertrophic scar fibroblasts	Zhu et al., 2015 [131]
	Cell cycle Invasion Differentiation	Human	Colorectal cancer tissue and colorectal cancer cell lines	Panza et al., 2014 [96]

miR-223	Inflammation	Mouse	Bone marrow derived macrophages Adipocyte-derived stromal cells Primary adipocytes	Ying et al., 2015 [133]

miR-329	Inflammation Oxidative stress	Rat	Cerebral cortex, Pheochromocytoma cell line	Dharap et al., 2015 [17]

miR-339-5p	Adipocyte differentiation	Human	White adipose tissue Isolated preadipocytes	Yu et al. 2014 [134]

miR-378	Adipocyte differentiation	Human	White adipose tissue Primary adipose derived stromal cells	Yu et al., 2014 [134]

## Abbreviations

AP-1: Activator protein-1
ApoA: Apolipoprotein-A
BAT: Brown adipose tissue
CD36: Cluster of differentiation 36
FAT: Fatty acid translocase
C/EBP*α*: CCAAT/enhancer-binding protein *α*
C. elegans: Caenorhabditis elegans
COX-2: Cyclooxygenase-2
CRC: Colorectal carcinoma
CREB: cAMP response element-binding protein
CVD: Cardiovascular disease
DEN: Diethylnitrosamine
DGCR8: DiGeorge syndrome chromosomal region 8
ECM: Extracellular matrix
FABP: Fatty acid binding protein
Glut4: Glucose-transporter 4
HCC: Hepatocellular carcinoma
HCV/HBV: Hepatitis C/B virus
HFD: High fat diet
HSC: Hepatic stellate cells
IRS-1/2: Insulin receptor substrates-1/2
lncRNA: Long noncoding RNA
LPL: Lipoprotein lipase
LPS: Lipopolysaccharide
LRH-1: Liver receptor homolog-1
MCP-1: Monocyte chemoattractant protein-1
miRNA: MicroRNA
MSC: Mesenchymal stem cell
NAFLD: Nonalcoholic fatty liver disease
NASH: Nonalcoholic steatohepatitis
NCOR: Nuclear receptor corepressor
ncRNA: Noncoding RNA
NF*κ*B: Nuclear factor-*κ*B
PGE2: Prostaglandin E2
PGC1: Peroxisome proliferator-activated receptor gamma coactivator-1
PPAR: Peroxisome proliferator-activated receptor
PPRE: Peroxisome proliferator response element
Prdm16: PR domain-containing 16
RISC: RNA-induced silencing complex
ROS: Reactive oxygen species
RXR: Retinoid-X-Receptor
siRNA: Small interfering RNA
Sirt1: Sirtuin 1
SMRT: Silencing mediator for retinoid and thyroid hormone receptor
sncRNA: Small noncoding RNA
STAT1: Signal transducer and activator of transcription 1
T2DM: Type 2 diabetes mellitus
TARP2: T-cell receptor gamma-chain constant region
TNF-*α*: Tumor necrosis factor-*α*
TZD: Thiazolidinedione
UCP-1: Uncoupling protein-1
UTR: Untranslated region
VCAM: Vascular cell adhesion protein
WAT: White adipose tissue.
